# Efficacy and Safety of the Melphalan/Hepatic Delivery System in Patients with Unresectable Metastatic Uveal Melanoma: Results from an Open-Label, Single-Arm, Multicenter Phase 3 Study

**DOI:** 10.1245/s10434-024-15293-x

**Published:** 2024-05-04

**Authors:** Jonathan S. Zager, Marlana Orloff, Pier Francesco Ferrucci, Junsung Choi, David J. Eschelman, Evan S. Glazer, Aslam Ejaz, J. Harrison Howard, Erika Richtig, Sebastian Ochsenreither, Sunil A. Reddy, Michael C. Lowe, Georgia M. Beasley, Anja Gesierich, Armin Bender, Martin Gschnell, Reinhard Dummer, Michel Rivoire, Ana Arance, Stephen William Fenwick, Joseph J. Sacco, Sebastian Haferkamp, Carsten Weishaupt, Johnny John, Matthew Wheater, Christian H. Ottensmeier

**Affiliations:** 1 Departments of Cutaneous Oncology and Sarcoma, Moffitt Cancer Center, Tampa, FL USA; 2 Department of Oncologic Sciences, University of South Florida Morsani College of Medicine, Tampa, FL USA; 3 Thomas Jefferson University, Philadelphia, PA USA; 4 European Institute of Oncology, IRCCS, Milan, Italy; 5 The University of Tennessee Health Science Center, Memphis, TN USA; 6 The Ohio State University, Columbus, OH USA; 7 University of South Alabama, Mobile, AL USA; 8 Medical University of Graz, Graz, Austria; 9 Charité Comprehensive Cancer Center, Berlin, Germany; 10 Stanford University, Stanford, CA USA; 11 Emory University, Atlanta, GA USA; 12 Duke University, Durham, NC USA; 13 University Hospital Würzburg, Würzburg, Germany; 14 University Hospital Marburg, Marburg, Germany; 15 University Hospital Zürich, Zürich, Switzerland; 16 Léon Bérard Center, Lyon, France; 17 Hospital Clínic Barcelona, Barcelona, Spain; 18 Liverpool University Hospitals NHS Foundation Trust, Liverpool, UK; 19 The Clatterbridge Cancer Center, University of Liverpool, Liverpool, UK; 20 University Hospital Regensburg, Regensburg, Germany; 21 University Hospital Münster, Münster, Germany; 22 Delcath Systems, Inc., Queensbury, NY USA; 23 University Hospital Southampton NHS Foundation Trust, Southampton, UK

## Abstract

**Background:**

Uveal melanoma (UM) has a poor prognosis once liver metastases occur. The melphalan/Hepatic Delivery System (melphalan/HDS) is a drug/device combination used for liver-directed treatment of metastatic UM (mUM) patients. The purpose of the FOCUS study was to assess the efficacy and safety of melphalan/HDS in patients with unresectable mUM.

**Methods:**

Eligible patients with mUM received treatment with melphalan (3.0 mg/kg ideal body weight) once every 6 to 8 weeks for a maximum of six cycles. The primary end point was the objective response rate (ORR). The secondary end points included duration of response (DOR), overall survival (OS), and progression-free survival (PFS).

**Results:**

The study enrolled 102 patients with mUM. Treatment was attempted in 95 patients, and 91 patients received treatment. In the treated population (*n* = 91), the ORR was 36.3 % (95 % confidence interval [CI], 26.44–47.01), including 7.7 % of patients with a complete response. Thus, the study met its primary end point because the lower bound of the 95 % CI for ORR exceeded the upper bound (8.3 %) from the benchmark meta-analysis. The median DOR was 14 months, and the median OS was 20.5 months, with an OS of 80 % at 1 year. The median PFS was 9 months, with a PFS of 65 % at 6 months. The most common serious treatment-emergent adverse events were thrombocytopenia (15.8 %) and neutropenia (10.5 %), treated mostly on an outpatient basis with observation. No treatment-related deaths were observed.

**Conclusion:**

Treatment with melphalan/HDS provides a clinically meaningful response rate and demonstrates a favorable benefit-risk profile in patients with unresectable mUM (study funded by Delcath; ClinicalTrials.gov identifier: NCT02678572; EudraCT no. 2015-000417-44).

**Supplementary Information:**

The online version contains supplementary material available at 10.1245/s10434-024-15293-x.

Uveal melanoma (UM) accounts for approximately 3 % to 5 % of all melanoma cases in the United States and represents the most common intraocular malignancy in adults.^1^ During its natural history, up to 50 % of patients with UM will eventually experience the development of metastatic disease, most often to the liver (~90 % of cases).^1^ The prognosis of metastatic UM (mUM) patients with hepatic metastases is dismal, with a median overall survival (mOS) of approximately 1 year.^2,3^

Treatment of mUM is challenging because, aside from tebentafusp (limited to HLA-A*02:01-positive patients), commonly used systemic therapies rarely produce durable responses or significant survival benefit.^1,4,5^ Immune checkpoint inhibitors (ICIs), including pembrolizumab, nivolumab and ipilimumab, have shown limited efficacy in mUM patients, with objective response rates (ORRs) for single-agent or dual immune checkpoint blockade (ICB) ranging from 0 to 16.7 % in retrospective chart analyses.^6–9^ The combination of nivolumab and ipilimumab showed an ORR of 18 % in a small, prospective single-center study^10^ and 11.5 % in a prospective multicenter study^11^ with mUM patients. A recent review^12^ reported overall ORR, median progression-free survival (mPFS), and mOS of 9.2 %, 3.0 months, and 11.5 months, respectively, for ICI in mUM.

Because the vast majority of patients with mUM will have liver metastases, often leading to liver failure,^13^ the National Comprehensive Cancer Network guidelines recommend for those patients, among other options, liver-directed therapies, including transarterial chemoembolization (TACE), radioembolization or immunoembolization, and thermal ablation, as well as locoregional perfusion procedures delivering high-dose chemotherapeutic agents, namely, intrahepatic perfusion (IHP), a surgical procedure, and percutaneous hepatic perfusion (PHP), a minimally invasive procedure.^14^

The PHP procedure requires the use of a hepatic delivery system (HDS), commercially available in Europe as CHEMOSAT (Delcath Systems, Inc., Queensbury, NY), and in the United States as the HEPZATO KIT (melphalan/HDS; Delcath Systems, Inc., Queensbury, NY), recently approved by the U.S. Food and Drug Administration (FDA) as the first approved liver-directed treatment for unresectable mUM patients.^15^

Ideally, a liver-directed treatment should treat the whole liver and target all radiographically evident and occult metastases, allow for retreatment for optimized tumor control, and achieve an acceptable benefit/risk profile. Radioembolization and TACE fulfill some of these requirements and generally achieve better control of hepatic metastases than systemic therapy. However, these procedures have limitations with respect to repeatability of treatment and ability to treat the whole liver.^13^ Although IHP treats the whole liver and has shown promising tumor response rates, its use is restricted by a high risk of morbidity and mortality due to its invasive nature. Most patients undergo only one treatment, which significantly limits its use and patient outcomes.^16,17^ The PHP procedure is a minimally invasive technique optimized to chemosaturate the whole liver without major surgery, and most patients are able to receive multiple treatments.

Results from early-phase clinical studies investigating the safety and efficacy of PHP using melphalan/HDS in mUM patients show encouraging signals of efficacy, including promising ORRs and OS relative to historic controls.^18–22^ The multicenter, open-label, phase 3 FOCUS study was designed to evaluate the efficacy and safety of melphalan/HDS in patients with unresectable mUM.

## Methods

### Patients

The study population included male and female patients 18 years of age or older with histologically verified mUM to the liver; liver tumor involvement of up to 50 %; at least one measurable liver lesion as assessed by contrast-enhanced computed tomography (CT) scans of the chest, abdomen, and pelvis as well as contrast-enhanced MRI of the liver (and brain and bone scans when indicated); and an Eastern Cooperative Oncology Group (ECOG)^23^ performance status of 0 to 1 at screening. Patients could be previously treated or treatment naïve, and could have limited extrahepatic disease amenable to resection or radiation.

The PHP procedure requires general anesthesia and active coagulation/anti-coagulation control. The eligibility criteria were designed to minimize the risks associated with the procedure (e.g., exclusion of patients with moderate or severe liver cirrhosis, portal hypertension, or New York Heart Association [NYHA] II–IV status). The eligibility criteria remained unchanged throughout the study and are provided in the study protocol. Detailed inclusion and exclusion criteria are mentioned in the Supplementary Appendix.

### Study Design and Treatment

The FOCUS study, conducted at 23 centers across the United States and Europe, was initiated as a two-arm, controlled, randomized study. Eligible patients were randomized 1:1 to receive melphalan/HDS or best alternative care (BAC) including investigator’s choice of TACE, pembrolizumab, ipilimumab, or dacarbazine. Due to slow enrollment, with patient reluctance to receive BAC treatment, the study design was amended to a single-arm study, after which all eligible patients received treatment with melphalan/HDS.

The patients received melphalan treatment (3.0 mg/kg ideal body weight; maximum dose of 220 mg for a single treatment) once every 6 to 8 weeks for a maximum of six cycles. Before each treatment, liver venous outflow was isolated by a double-balloon catheter placed into the inferior vena cava. Melphalan was administered for 30 min via an infusion catheter placed in the hepatic artery. The infusion was followed by 30 min of washout with extracorporeal filtration to further reduce systemic exposure to melphalan. All the patients received granulocyte colony-stimulating factor within 72 h after each PHP procedure. Treatment procedures were administered by a team of medical or surgical oncology, interventional radiology, and anesthesiology personnel, and a perfusionist.

All lesions were assessed with follow-up imaging performed every 12 weeks until disease progression using the same techniques as at baseline. Further details are included in the Supplementary Appendix.

### End Points and Assessments

The primary end point of the study was the objective response rate (ORR), as determined by the Independent Review Committee (IRC) based on Response Evaluation Criteria in Solid Tumors (RECIST) version 1.1.^24^ The secondary end points were duration of response (DOR), progression-free survival (PFS), overall survival (OS), and disease control rate (DCR).

Adverse events (AEs) were assessed by investigators and graded according to National Cancer Institute Common Terminology Criteria for Adverse Events version 4.03.^25^ Post hoc analyses were performed to evaluate the relationship between tumor response and survival and to assess outcomes for the patients with hepatic-only disease versus hepatic and extrahepatic disease.

### Study Oversight

The sponsor and all the authors contributed to various elements of the study design, protocol development, and data analysis. The protocol was approved by the institutional review board or independent ethics committee at each study center, as well as by all relevant competent authorities. The FOCUS study was conducted in accordance with the principles of the Declaration of Helsinki and Good Clinical Practice guidelines as outlined by the International Council for Harmonization. An IRC and an independent data safety monitoring board provided determination of efficacy and oversight of safety, respectively. The IRC was composed of board-certified radiologists with extensive experience in oncology. Imaging was assessed by two independent readers. Any disagreement about a patient’s response to treatment was adjudicated by a third reader. All the participants provided written informed consent. All the authors vouch for the accuracy and completeness of the data and for the fidelity of the study to the protocol.

### Statistical Analysis

Once the study design was changed to a single-arm investigation, the primary end point was changed from OS to ORR in agreement with the FDA, and sample size re-estimation was implemented. A meta-analysis of historical data (16 publications including 476 patients who had mUM treated with monotherapy or combination systemic immunotherapy) was performed to establish an ORR benchmark (details are included in the Supplementary Appendix). Pooled rates across studies for ORR were calculated based on both a random-effects model and a fixed-effects model. Heterogeneity in effect size was formally examined using the *Q* statistic and the *I*^2^ statistic using Comprehensive Meta-Analysis software Version 2.2 (Biostat, Englewood, NJ). The pooled ORR estimate (a weighted mean of the observed ORR) was 5.5 % (95 % confidence interval [CI], 3.6–8.3 %). The study was powered to test whether the lower bound of the 95 % CI for ORR would exceed the upper bound (8.3 %) of the meta-analysis. The new sample size was estimated to provide a maximum width of the 95 % CI of ±11.3 % around the point estimate of ORR, assuming that ORR was 21 %.

Efficacy analyses involved all patients treated with melphalan/HDS during both the randomized and single-arm phases of the study. The primary efficacy end point, ORR, was determined for all the patients treated with melphalan/HDS. Unless specified otherwise, statistical testing used a two-sided test at the 0.05 significance level. Nominal *p* values were reported without control for study-wide type 1 error because analyses were exploratory. Demographic data were summarized using descriptive statistics. Summary statistics for continuous variables include mean, standard deviation, median, and range (minimum to maximum). Categorical variables are presented as frequency counts and percentages.

Time-to-event variables were summarized using Kaplan-Meier methods. For the calculation of time-to-event end points except for DOR, the start date was the patient eligibility date. Post hoc analysis was performed to compare OS by best overall response. Descriptive statistics were used for safety analysis in the safety population, which included all patients for whom a study treatment or procedure was attempted. No statistical testing was applied to the exploratory efficacy analyses. Analyses were performed using SAS/STAT software, Version 9.4 of the SAS System for Windows (SAS Institute Inc., Cary, NC).

## Results

### Patient Disposition and Baseline Characteristics

From February 2016 to October 2020, 102 patients were enrolled and assigned to the melphalan/HDS group (pooled from the randomized and non-randomized portions of the study). Of the 102 patients, 95 had at least one PHP procedure initiated (safety population), and 91 received treatment with melphalan/HDS (treated population).

More than one third (37.4 %) of the patients in the treated population completed the maximum of six cycles permitted per protocol. The primary reasons for discontinuation were disease progression (28.6 %) and AEs/serious AEs (18.7 %). At the time of the data cutoff (2 December 2022), the median follow-up period was 36.4 months, and 17.6 % of the treated patients were still being followed for survival. In the treated population, the median time from diagnosis of primary tumor to study entry was 39.3 months (range, 0.7–198.9 months), and the median time from diagnosis of metastatic disease to study entry was 5.5 months (range, 0.2–67.5 months) (Table 1). The liver metastasis stage distribution was 38.5 % for stage M1a mUM, 56.0 % for stage M1b mUM, and 5.5 % for stage M1c mUM.^26^ The majority of the patients (87.9 %) had an ECOG performance status score of 0 (Table 1). Of the treated population, 44 % had received prior therapy for mUM (Table 1).

**Table 1:** Patient demographics, baseline characteristics, and exposure to study treatment for patients treated with melphalan/Hepatic Delivery System (treated population)

Characteristic	Melphalan/HDS (*n* = 91) *n* (%)
*Demographics and baseline characteristics*	
Median age: years (range)	61.0 (20–78)
Male sex	44 (48.4)
Ethnicity	
Hispanic or Latino	2 (2.2)
Non-Hispanic or Latino	86 (94.5)
No response	3 (3.3)
Race	
White	86 (94.5)
Other	2 (2.2)
No response	3 (3.3)
Median time since primary diagnosis: months (range)^a^	39.3 (0.7–198.9)
Median time since diagnosis of liver metastases: months (range)^a^	5.5 (0.2–67.5)
Median time from primary diagnosis to metastasis to liver: months (range)	33.0 (0.0–159.1)
ECOG performance status score	
0	80 (87.9)
1	9 (9.9)
Not recorded	2 (2.2)
Elevated LDH	32/86 (37.2)
Extent of liver involvement (%)^b^	
≤25	72 (79.1)
26−50	19 (20.9)
Largest hepatic lesion^c,d^	
≤ 3 cm (stage M1a)	35 (38.5)
3.1 to 8 cm (stage M1b)	51 (56.0)
≥ 8.1 cm (stage M1c)	5 (5.5)
Extrahepatic lesions^d^	
Lung	11/27 (40.7)
Lymph node	5/27 (18.5)
Bone^e^	4/27 (14.8)
Soft tissue^f^	10/27 (37.0)
Brain	0/27 (0.0)
Other visceral^g^	6/27 (22.2)
Prior therapies^h^	40 (44.0)
Radiation	10 (11.0)
Surgery^i^	13 (14.3)
Systemic	23 (25.3)
Immune checkpoint inhibitor	21 (23.1)
Chemotherapy	3 (3.3)
SIRT	1 (1.1)
TACE	1 (1.1)
Targeted small molecule	1 (1.1)
*Exposure to study treatment*	
No. of treatment cycles completed	
1	7 (7.7)
2	18 (19.8)
3	11 (12.1)
4	15 (16.5)
5	6 (6.6)
6	34 (37.4)

HDS, hepatic delivery system; ECOG, Eastern Cooperative Oncology Group; LDH, lactate dehydrogenase; SIRT, Selective internal radiation therapy; TACE, transarterial chemoembolization

^a^Months from diagnosis of either primary tumor or liver metastases to either randomization (double-arm phase) or eligibility (single-arm phase)

^b^Assessed by the investigator

^c^Tumor staging per *AJCC Cancer Staging Manual,*7th edition

^d^Based on Independent Review Committee assessment

^e^Includes spine, lumbar spine, pelvis, ribs, sacrum, and skull

^f^Includes subcutaneous trunk and chest wall

^g^Includes spleen and adrenal gland

^h^Patients with multiple therapies of a given type are counted once for that type

^i^Includes only therapeutic surgeries/procedures and excludes non-therapeutic prior surgeries/procedures (e.g., biopsy). Each surgery/procedure was retrospectively classified as therapeutic or non-therapeutic

### Efficacy

In the treated population, the primary end point, ORR, was 36.3 % (95 % CI, 26.44–47.01 %), as assessed by IRC (Table 2). The best overall response was a complete response (CR) for 7.7 %, a partial response (PR) for 28.6 %, and stable disease (SD) for 37.4 % of the patients (Table 2). In the treated population, DCR was 73.6 % (95 % CI, 63.35–82.31 %).

**Table 2:** Clinical outcomes in patients treated with melphalan/Hepatic Delivery System (treated population, assessed by Independent Review Committee)

Characteristic	Melphalan/HDS (*n* = 91) *n* (%)
*Primary end point*	
Objective response rate: % (95 % CI)^a^	36.3 (26.44–47.01)
No. of patients who achieved objective response	33
Best overall response^b,c^	
Complete response	7 (7.7)
Partial response	26 (28.6)
Stable disease	34 (37.4)
Progressive disease	23 (25.3)
Not evaluable	1 (1.1)
*Secondary end points*	
Median duration of response in responders: months (95 % CI)^d^	14.0 (8.31–17.74); *n* = 33
Disease control rate: % (95 % CI)^a^	73.6 (63.35–82.31)
No. of patients who achieved disease control	67
Median progression-free survival: months (95 % CI)^d^	9.0 (6.34–11.56)
Progression-free survival at 6 months: % (95 % CI)^d^	65 (54–74)
Progression-free survival at 1 year: % (95 % CI)^d^	38 (27–48)
Median overall survival: months (95 % CI)^d^	20.5 (16.79–25.26)
Overall survival at 1 year: % (95 % CI)^d^	80 (70–87)
Overall survival at 2 years: % (95 % CI)^d^	43 (32–53)
*Exploratory end points*	
Median time to objective response: months (95 % CI)^d^	3.3 (2.86–5.59); *n* = 33
Median hepatic progression-free survival: months (95 % CI)^d^	13.9 (9.30–16.66)
Hepatic objective response rate: % (95 % CI)^a^	41.8 (31.50–52.57); *n* = 38

HDS, hepatic delivery system; CI, confidence interval

^a^Patients without at least 1 post-baseline response assessment were designated as non-responders

^b^Best overall response per Independent Review Committee (Response Evaluation Criteria in Solid Tumors v1.1) from the date of randomization/eligibility until disease progression

^c^For complete response or partial response, confirmation was required by repeat assessment ≥4 weeks after initial documentation. To qualify as stable disease, the image must have been taken at least 9 weeks after start of therapy

^d^Kaplan-Meier estimate

**Fig. 1:**
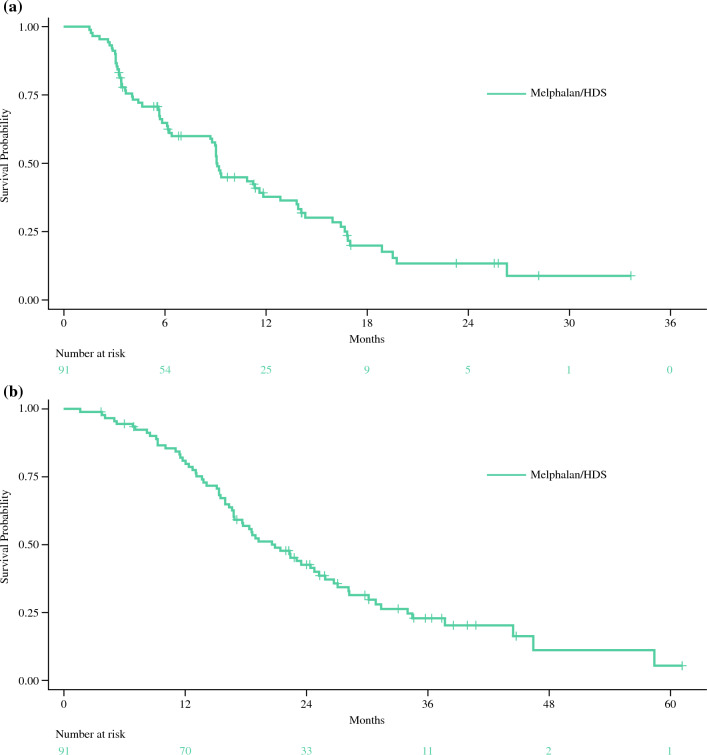
Kaplan-Meier plots of **a** progression-free survival and **b** overall survival in patients treated with melphalan/Hepatic Delivery System (HDS) (treated population: assessed by Independent Review Committee)

Responders had a median DOR of 14.0 months (95 % CI, 8.31–17.74 months; Table 2). The mPFS was 9 months (95 % CI, 6.34–11.56 months; Table 2; Fig. 1a). At the 2 December 2022 data cutoff, the median OS in the treated population was 20.5 months (95 % CI, 16.79–25.26 months), with 80 % of the patients surviving at least 1 year and 43 % of the patients surviving at least 2 years (Table 2; Fig. 1b). The median time to objective response and hepatic PFS were respectively 3.3 months (95 % CI, 2.86–5.59 months) and 13.9 months (95 % CI, 9.30–16.66 months). The hepatic ORR was 41.8 % (95 % CI, 31.50–52.57 %; Table 2).

**Fig. 2:**
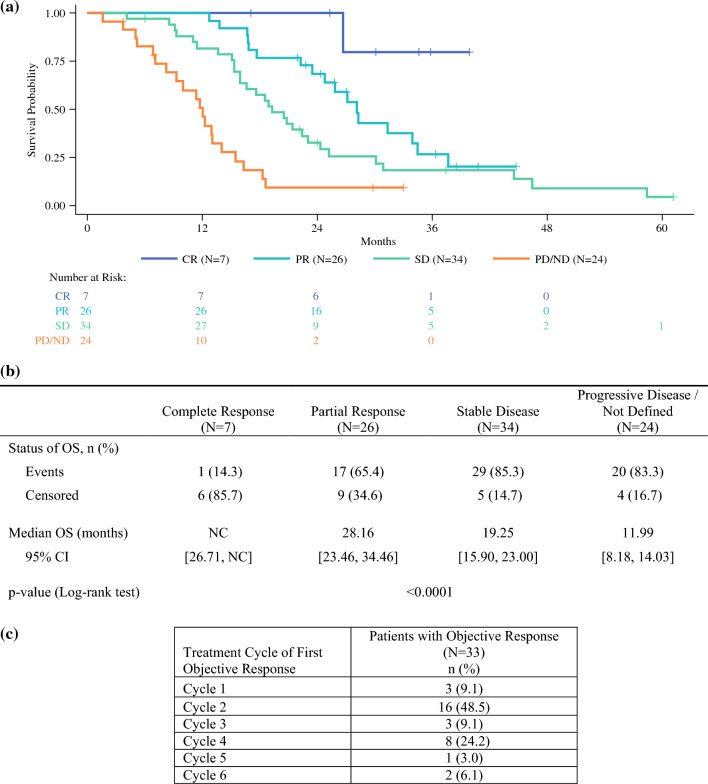
Post hoc analysis. **a** Kaplan-Meier plot of overall survival by best overall response. **b** Overall survival by best overall response and **c** first occurrence of objective response by treatment cycle in patients treated with the melphalan/Hepatic Delivery System (treated population, assessed by Independent Review Committee). CI, confidence interval; CR, complete response; NC, not calculable; ND, not defined; OS, overall survival; PD, progressive disease; PR, partial response; SD, stable disease

The results from a post hoc analysis of the relationship between tumor response and survival demonstrated a statistically significant difference (*P* < 0.0001) between the patients who had a best overall response of PR, SD, and progressive disease (PD), with respective median OS values of 28.2 months (95 % CI, 23.46–34.46 months), 19.3 months (95 % CI, 15.90–23.00 months), and 12.0 months (95 % CI, 8.18–14.03 months). The median OS could not be calculated for the patients with CR because only one event was observed before the data cutoff (Fig. 2a and b). The analysis of objective response by PHP treatment cycle showed that more than 50 % of all objective responses began after the first or second treatment cycle (Fig. 2c).

### Safety

Among the 95 patients assessed for safety after treatment with melphalan/HDS (including attempted treatments), all the patients reported at least one treatment-emergent AE (TEAE) (Table 3). The most common TEAEs (any grade) were thrombocytopenia (65.3 %), anemia (63.2 %), nausea (56.8 %), fatigue (53.7 %), leukopenia (46.3 %), neutropenia (34.7 %), vomiting (34.7 %), and alanine aminotransferase (ALT) increased (31.6 %) (Table 3). Grades 3 and 4 TEAEs were reported in 81.1 % of the patients including grade 3 or 4 thrombocytopenia (54.7 %), leukopenia (33.7 %), anemia (32.6 %), and neutropenia (29.5 %) (Table 3). The most common serious TEAEs reported were thrombocytopenia (15.8 %) and neutropenia (10.5 %) (Table 3). Three patients died during the study. The causes of death were cardiac arrest, acute hepatic failure, and bacterial peritonitis, occurring at 43, 62, and 64 days, respectively, after the last study treatment. None of the three deaths were considered related to study treatment, device, or procedure. Treatment was discontinued for 17.9 % of the patients due to TEAEs (Table 3).

**Table 3:** Treatment-emergent adverse events in patients treated with melphalan/Hepatic Delivery System (safety population)^a^

Parameters	Melphalan/HDS (*n* = 95) *n* (%)	
Any TEAE leading to discontinuation of study treatment	17 (17.9)	
Any TEAE leading to dose reduction of study treatment	13 (13.7)	
Death	3 (3.2)	

	Any grade	Grade 3/4
*Any TEAE*	95 (100.0)	77 (81.1)
Thrombocytopenia^b^	62 (65.3)	52 (54.7)
Anemia^c^	60 (63.2)	31 (32.6)
Nausea	54 (56.8)	0 (0.0)
Fatigue	51 (53.7)	0 (0.0)
Leukopenia^d^	44 (46.3)	32 (33.7)
Neutropenia^e^	33 (34.7)	28 (29.5)
Vomiting	33 (34.7)	0 (0.0)
ALT increased	30 (31.6)	3 (3.2)
INR increased	29 (30.5)	8 (8.4)
Activated PTT prolonged	27 (28.4)	8 (8.4)
AST increased	27 (28.4)	4 (4.2)
Blood alkaline phosphatase increased	26 (27.4)	2 (2.1)
Back pain	25 (26.3)	1 (1.1)
Dyspnea	22 (23.2)	2 (2.1)
Abdominal pain upper	21 (22.1)	1 (1.1)
Headache	18 (18.9)	0 (0.0)
Abdominal pain	16 (16.8)	0 (0.0)
Contusion	16 (16.8)	0 (0.0)
Diarrhea	15 (15.8)	1 (1.1)
Decreased appetite	15 (15.8)	0 (0.0)
Pyrexia	15 (15.8)	0 (0.0)
Cough	14 (14.7)	0 (0.0)
Hypocalcemia	12 (12.6)	3 (3.2)
Troponin I increased	12 (12.6)	2 (2.1)
Asthenia	12 (12.6)	0 (0.0)
Hypotension	11 (11.6)	3 (3.2)
Lethargy	11 (11.6)	0 (0.0)
Blood bilirubin increased	10 (10.5)	3 (3.2)
Groin pain	10 (10.5)	0 (0.0)
Pain in extremity	10 (10.5)	0 (0.0)
Hypophosphatemia	9 (9.5)	7 (7.4)
Febrile neutropenia	8 (8.4)	7 (7.4)
*Any serious TEAE*	43 (45.3)	38 (40.0)
Thrombocytopenia^b^	15 (15.8)	15 (15.8)
Neutropenia^e^	10 (10.5)	10 (10.5)
Febrile neutropenia	7 (7.4)	6 (6.3)
Leukopenia^d^	5 (5.3)	5 (5.3)

HDS, hepatic delivery system; TEAE, treatment-emergent adverse event; ALT, alanine aminotransferase; INR, international normalized ratio; PTT, partial thromboplastin time; AST, aspartate aminotransferase

^a^TEAEs reported in at least 10 % of patients (any grade) or in at least 5 % of patients (grade 3/4 and serious TEAEs) treated with melphalan/Hepatic Delivery System

^b^Thrombocytopenia includes thrombocytopenia and platelet count decreased

^c^Anemia includes anemia, febrile bone marrow aplasia, anemia, normochromic normocytic anemia, and red blood cell count decreased

^d^Leukopenia includes leukopenia, lymphocyte count decreased, lymphopenia, and white blood cell count decreased

^e^Neutropenia includes neutropenia and neutrophil count decreased

Peri-procedural TEAEs that occurred in 20 % or more of the patients were anemia (56.0 %), thrombocytopenia (50.5 %), nausea (41.8 %), international normalized ratio (INR) increased (30.8 %), vomiting (29.7 %), prolonged activated partial thromboplastin time (PTT) (28.6 %), fatigue (23.1 %), aspartate aminotransferase (AST) increased (23.1 %), and ALT increased (22.0 %) (Table 4).

**Table 4:** Peri-procedural treatment-emergent adverse events in patients treated with melphalan/Hepatic Delivery System (treated population)^a^

Parameters	Melphalan/HDS (*n* = 91) *n* (%)	
	Any grade	Grade 3/4
*Any peri-procedural TEAE*	80 (87.9)	51 (56.0)
Anemia^b^	51 (56.0)	27 (29.7)
Thrombocytopenia^c^	46 (50.5)	35 (38.5)
Nausea	38 (41.8)	0 (0.0)
INR increased	28 (30.8)	8 (8.8)
Vomiting	27 (29.7)	0 (0.0)
Activated PTT prolonged	26 (28.6)	8 (8.8)
AST increased	21 (23.1)	3 (3.3)
Fatigue	21 (23.1)	0 (0.0)
ALT increased	20 (22.0)	1 (1.1)
Leukopenia^d^	17 (18.7)	12 (13.2)
Back pain	15 (16.5)	1 (1.1)
Hypocalcemia	12 (13.2)	3 (3.3)
Troponin I increased	12 (13.2)	2 (2.2)
Contusion	10 (11.0)	0 (0.0)
Pyrexia	10 (11.0)	0 (0.0)
Hypophosphatemia	8 (8.8)	7 (7.7)
*Any serious TEAE*	20 (22.0)	16 (17.6)
Thrombocytopenia^c^	5 (5.5)	5 (5.5)

HDS, hepatic delivery system; TEAE, treatment-emergent adverse event; INR, international normalized ratio; PTT, partial thromboplastin time; AST, aspartate aminotransferase; ALT, alanine aminotransferase

^a^Peri-procedural TEAEs reported in at least 10 % of patients (any grade) or in at least 5 % of patients (grade 3/4 and serious TEAEs) in patients treated with melphalan/Hepatic Delivery System. TEAEs with onset from the procedure date to the earlier of discharge from hospital or day 3 were considered peri-procedural

^b^Anemia includes anemia, febrile bone marrow aplasia, anemia, normochromic normocytic anemia, and red blood cell count decreased

^c^Thrombocytopenia includes thrombocytopenia and platelet count decreased

^d^Leukopenia includes leukopenia, lymphocyte count decreased, lymphopenia, and white blood cell count decreased

## Discussion

Metastatic uveal melanoma remains a difficult-to-treat disease with limited therapeutic options. Tebentafusp, a bispecific immunotherapeutic agent, is indicated for HLA-A*02:01-positive adult patients with unresectable mUM^27^ and represents a treatment option for approximately 45 % of mUM patients who are HLA-A*02:01-positive.^5^ The HEPZATO KIT, recently approved by the FDA based on the results from the FOCUS study, is the only FDA-approved liver-directed treatment for patients with mUM and is not limited by tumor genotype, thus offering broad utility for this indication.

The FOCUS study evaluated PHP using the drug/device combination of melphalan/HDS for treatment of patients with unresectable mUM. This approach enables locoregional delivery of a high melphalan dose to the liver and minimizes systemic exposure and melphalan-related AEs with the use of active filters to remove excess melphalan after liver perfusion.

The study population was heterogeneous and included patients with hepatic-only disease and those with hepatic and limited extrahepatic disease, patients with up to 50 % of liver tumor involvement (79.1 % of patients had ≤25 % liver tumor burden), and both previously treated (44.0 %) and treatment-naïve (56.0 %) patients. The diverse study population together with operational performance at 23 study centers enabled a robust evaluation of the efficacy and safety of melphalan/HDS in patients with unresectable mUM.

The primary end point of the study was met by a wide margin. With an ORR of 36.3 %, as assessed by IRC, the lower bound of the 95 % CI for ORR (26.4 %) was well above the upper bound from the benchmark meta-analysis (8.3 %). Efficacy of PHP was consistent for patients with both hepatic-only and hepatic and extrahepatic disease, including ORRs of 37.5 % and 33.3 %, respectively, and mPFS periods of 9.3 and 6.2 months, respectively. These results compare favorably with the overall ORR of 11.5 % and mPFS of 1.5 months for hepatic-only disease versus 3.7 months for hepatic and extrahepatic disease reported in treatment-naïve mUM patients receiving nivolumab plus ipilimumab.^11^

The efficacy of melphalan/HDS against extrahepatic lesions may be, at least in part, explained by residual melphalan systemic exposure, estimated at 14 % of the administered dose. In the FOCUS study, mOS also was consistent in patients with hepatic-only disease (20.8 months) and hepatic and extrahepatic disease (18.9 months). This consistency contrasts with mOS results seen with nivolumab plus ipilimumab treatment, which suggest shorter survival in patients with hepatic-only mUM at a median of 9.2 versus 15.5 months for patients with hepatic and extrahepatic disease.^11^

The mOS of 20.5 months compares favorably with results from meta-analyses of survival rates across various treatments, including chemotherapy, systemic ICI, and liver-directed therapy, which range from 10.2 to 12.8 months across all therapies.^2,3^ In the IMCgp100-202 study^5^, the mOS was 21.7 months in the tebentafusp arm and 16 months in the control arm. The mPFS of 9.0 months also compares favorably with the mPFS of 3.3 months from a meta-analysis of various treatments^3^ and with the mPFS from the IMCgp100-202 study of 3.3 months in the tebentafusp group and 2.9 months in the control group.

Comparisons with results from other clinical studies evaluating liver-directed therapies in mUM patients are difficult given markedly different patient populations (e.g., exclusion of patients with extrahepatic disease and other methodologic differences). The EORTC 18021 study of treatment-naïve patients compared efficacy and safety of fotamustine administered intravenously or via hepatic intra-arterial (HIA) infusion. In the HIA arm, mOS was 14.6 months, mPFS was 4.5 months, and ORR was 10.5 %.^28^ In a double-blind, randomized phase 2 study, mOS was 21.5 months, mPFS was 10.4 months, and ORR was 21.2 %.^29^

The safety profile of melphalan/HDS in the FOCUS study was characterized mainly by hematologic toxicity due to systemic exposure to residual melphalan. Melphalan/HDS patients receive high doses of melphalan locoregionally (up to 220 mg per treatment), and the perfusion system filters remove up to 86 % of the administered melphalan dose.^30^ In the FOCUS study, as expected with the resultant level of systemic melphalan exposure, a majority of the patients experienced severe myelosuppression. The observed safety profile was consistent with previous experience at these exposure levels.^31,32^

The hematologic AEs were generally transient in nature, with nadirs at the end of the second week after each treatment, and were treated mostly on an outpatient basis with observation. Tolerability of melphalan/HDS was good, as evidenced by a median of four completed treatment cycles and nearly 40 % of the patients completing the planned six treatment cycles as per study protocol. Analysis of safety data by PHP treatment cycle did not indicate cumulative toxicity. Analysis of efficacy by treatment cycle indicated that more than 50 % of the objective tumor responses had onset after the first or second treatment cycle. No new safety signals for melphalan/HDS were reported.

An area of ongoing investigation is the combination of melphalan/HDS treatment with nivolumab and ipilimumab in mUM patients. Recently published results from a small phase 1b clinical study show a good safety profile for this combination as well as an intriguing early activity signal (DCR 100 %, ORR 86 %).^33^ The rationale for this combination regimen is based on the ability of melphalan/HDS to enhance antigen presentation by killing cancer cells, resulting in immunomodulation, whereas anti–CTLA-4 and anti–PD-1/PD/L1 antibodies enhance immune responses to weak tumor antigens and activation of tumor-reactive immune cells.

Melphalan/HDS is a promising liver-directed treatment option for unresectable mUM. Additional clinical studies of other tumor types with hepatic metastases and combinations with immunotherapy are needed to further explore the full clinical potential of this novel treatment approach.

## Conclusions

The FOCUS study provides robust evidence of the clinical benefit of melphalan/HDS for patients with unresectable mUM. This therapy offers a potential treatment option for patients with this rare indication, which is associated with a poor prognosis and has limited treatment options. Overall, the results demonstrated a favorable benefit-risk profile of melphalan/HDS for this patient population.

### Supplementary Information

Below is the link to the electronic supplementary material.

Supplementary file1 (DOCX 43 KB)
