# Correction: Efficacy and Safety of the Melphalan/Hepatic Delivery System in Patients with Unresectable Metastatic Uveal Melanoma: Results from an Open-Label, Single-Arm, Multicenter Phase 3 Study

**DOI:** 10.1245/s10434-024-15886-6

**Published:** 2024-07-18

**Authors:** Jonathan S. Zager, Marlana Orloff, Pier Francesco Ferrucci, Junsung Choi, David J. Eschelman, Evan S. Glazer, Aslam Ejaz, J. Harrison Howard, Erika Richtig, Sebastian Ochsenreither, Sunil A. Reddy, Michael C. Lowe, Georgia M. Beasley, Anja Gesierich, Armin Bender, Martin Gschnell, Reinhard Dummer, Michel Rivoire, Ana Arance, Stephen William Fenwick, Joseph J. Sacco, Sebastian Haferkamp, Carsten Weishaupt, Johnny John, Matthew Wheater, Christian H. Ottensmeier

**Affiliations:** 1 Departments of Cutaneous Oncology and Sarcoma, Moffitt Cancer Center, Tampa, FL USA; 2 Department of Oncologic Sciences, University of South Florida Morsani College of Medicine, Tampa, FL USA; 3 Thomas Jefferson University, Philadelphia, PA USA; 4 European Institute of Oncology, IRCCS, Milan, Italy; 5 The University of Tennessee Health Science Center, Memphis, TN USA; 6 The Ohio State University, Columbus, OH USA; 7 University of South Alabama, Mobile, AL USA; 8 Medical University of Graz, Graz, Austria; 9 Charité Comprehensive Cancer Center, Berlin, Germany; 10 Stanford University, Stanford, CA USA; 11 Emory University, Atlanta, GA USA; 12 Duke University, Durham, NC USA; 13 University Hospital Würzburg, Würzburg, Germany; 14 University Hospital Marburg, Marburg, Germany; 15 University Hospital Zürich, Zürich, Switzerland; 16 Léon Bérard Center, Lyon, France; 17 Hospital Clínic Barcelona, Barcelona, Spain; 18 Liverpool University Hospitals NHS Foundation Trust, Liverpool, UK; 19 The Clatterbridge Cancer Center, University of Liverpool, Liverpool, UK; 20 University Hospital Regensburg, Regensburg, Germany; 21 University Hospital Münster, Münster, Germany; 22 Delcath Systems, Inc., Queensbury, NY USA; 23 University Hospital Southampton NHS Foundation Trust, Southampton, UK

**Correction to: Ann Surg Oncol (2024) 31:5340–5351** 10.1245/s10434-024-15293-x

The article “Efficacy and Safety of the Melphalan/Hepatic Delivery System in Patients with Unresectable Metastatic Uveal Melanoma: Results from an Open-Label, Single-Arm, Multicenter Phase 3 Study ”, written by Zager et al. was originally published electronically on the publisher’s internet portal on May 4, 2024, without open access. With the authors' decision to opt for Open Choice the copyright of the article changed on July 14, 2024, to © The Author(s) 2024, and the article is forthwith distributed under a Creative Commons Attribution 4.0 International License, which permits use, sharing, adaptation, distribution and reproduction in any medium or format, as long as you give appropriate credit to the original author(s) and the source, provide a link to the Creative Commons licence, and indicate if changes were made. The images or other third party material in this article are included in the article’s Creative Commons licence, unless indicated otherwise in a credit line to the material. If material is not included in the article’s Creative Commons licence and your intended use is not permitted by statutory regulation or exceeds the permitted use, you will need to obtain permission directly from the copyright holder. To view a copy of this licence, visit http://creativecommons.org/licenses/by/4.0.

