# Supplementary material for: Efficacy and Safety of the Melphalan/Hepatic Delivery System in Patients with Unresectable Metastatic Uveal Melanoma: Results from an Open-Label, Single-Arm, Multicenter Phase 3 Study
Source: Ann Surg Oncol. 2024 May 4;31(8):5340–51. doi: 10.1245/s10434-024-15293-x (PMC11249544; doi:10.1245/s10434-024-15293-x)
Supplement: Supplementary file 1 — Supplementary file1 (DOCX 43 KB) [file 10434_2024_15293_MOESM1_ESM.docx]

# **Efficacy and Safety of the Melphalan/Hepatic Delivery System in Patients with Unresectable Metastatic Uveal Melanoma: Results from an Open-Label, Single-Arm, Multicenter Phase 3 Study**

Jonathan S. Zager, MD, FACS^1,2^; Marlana Orloff, MD^3^; Pier Francesco Ferrucci, MD^4^; Junsung Choi, MD^1,2^; David J. Eschelman, MD, FSIR^3^; Evan S. Glazer, MD, PhD, FACS^5^; Aslam Ejaz, MD^6^; J. Harrison Howard, MD^7^; Erika Richtig, MD^8^; Sebastian Ochsenreither, MD^9^; Sunil A. Reddy, MD^10^; Michael C. Lowe, MD^11^; Georgia M. Beasley, MD^12^; Anja Gesierich, MD^13^; Armin Bender, MD^14^; Martin Gschnell, MD^14^; Reinhard Dummer, MD^15^; Michel Rivoire, MD^16^; Ana Arance, MD, PhD^17^; Stephen William Fenwick, MD, FRCS^18^; Joseph J. Sacco, MD^19^; Sebastian Haferkamp, MD^20^; Carsten Weishaupt, MD^21^; Johnny John, MD^22^; Matthew Wheater, MD, PhD, MRCP^23^; Christian H. Ottensmeier, MD PhD, FRCP^18^

Affiliations:

*^1^Moffitt Cancer Center, Tampa, FL, USA; ^2^University of South Florida Morsani College of Medicine, Tampa, FL, USA; ^3^Thomas Jefferson University, Philadelphia, PA, USA; ^4^European Institute of Oncology, IRCCS, Milan, Italy; ^5^The University of Tennessee Health Science Center, Memphis, TN, USA; ^6^The Ohio State University, Columbus, OH, USA; ^7^* *University of South Alabama, Mobile, AL, USA*; ^8^*Medical University of Graz, Graz, Austria; ^9^Charité Comprehensive Cancer Center, Berlin, Germany; ^10^Stanford University, Stanford, CA, USA; ^11^Emory University, Atlanta, GA, USA; ^12^Duke University, Durham, NC, USA; ^13^University Hospital Würzburg, Würzburg, Germany; ^14^University Hospital Marburg, Marburg, Germany; ^15^University Hospital Zürich, Zürich, Switzerland; ^16^Léon Bérard Center, Lyon, France;* *^17^Hospital Clínic Barcelona, Barcelona, Spain; ^18^Liverpool University Hospitals NHS Foundation Trust, Liverpool, UK; ^19^The Clatterbridge Cancer Center, University of Liverpool, Liverpool, UK; ^20^University Hospital Regensburg, Regensburg, Germany; ^21^ University Hospital Münster, Münster, Germany;* ^22^*Delcath Systems, Inc., Queensbury, NY, USA; ^23^University Hospital Southampton NHS Foundation Trust, Southampton, UK.*

# SUPPLEMENTARY APPENDIX

- Figure S1. Participant Flow Diagram
- Inclusion/exclusion criteria
- Study treatment: melphalan/Hepatic Delivery System (melphalan/HDS)
- Dose and schedule of melphalan/HDS treatment
- Meta-analysis of historical data and sample size considerations

**References**

**Figure S1. Participant Flow Diagram**

**Melphalan/HDS**

- Enrolled: 102
- Received treatment: 91
- Did not receive treatment: 11
  - Treatment attempted: 4
  - Treatment not attempted: 7
- Completed the maximum 6 cycles of treatment permitted per protocol: 34
- Discontinued treatment: 57
  - Disease progression – Investigator decision: 15
  - Serious adverse event: 10
  - Investigator’s decision: 9
  - Disease progression requiring systemic therapy: 8
  - Adverse event: 6
  - Hepatic-only disease progression: 3
  - Withdrawal of consent: 3
  - COVID: 1
  - Poor patient compliance: 1
  - Serious adverse event resulting in death: 1

Treatment

Follow-up

Analysis population

- Intention-to-treat population N=102
- Safety population N=95
- Treated population N=91

### ***Inclusion/exclusion criteria***

*Inclusion criteria*

Subjects were to meet all of the following criteria for study entry:

- Male or female subjects ≥18 years of age.
- Subjects must weigh ≥35 kg (due to possible size limitations with respect to percutaneous catheterization of the femoral artery and vein using the Delcath Hepatic Delivery System [HDS]).
- 50% or less histologically or cytologically proven uveal melanoma (UM) metastases in the parenchyma of the liver.
- Disease in the liver measurable by computed tomography (CT) scan and/or magnetic resonance imaging (MRI).
- Evidence of limited extrahepatic disease on preoperative radiological studies was acceptable if the life-threatening component of disease was in the liver. Limited extrahepatic disease was defined as follows: metastasis in bone, subcutaneous, lung, or lymph nodes that was amenable to resection or radiation and had a defined treatment plan.
- Scans used to determine eligibility (CT scan of the chest/abdomen/pelvis and MRI of the liver) performed within 28 days prior to eligibility. An MRI of the liver was required at Screening to validate that CT scan accurately reflects the extent of disease in the liver. For subjects with MRI intolerance, a 3-phase liver CT scan was to be done in place of liver MRI.
- Subjects with no chemotherapy, radiotherapy, chemoembolization, radioembolization, or immunoembolization for their malignancy within 30 days prior to treatment and recovered from all side effects of therapeutic and diagnostic interventions except those listed in Appendix B of the study protocol.
- Subjects receiving anti-programmed cell death protein 1 immunotherapy, such as pembrolizumab or nivolumab, or human cytotoxic T-lymphocyte antigen 4 blocking antibody, such as ipilimumab, were to have completed treatment 8 weeks prior to study enrolment.
- Subjects with an Eastern Cooperative Oncology Group Performance Status (ECOG PS) score of 0 to 1 at Screening.
- Subjects with adequate hepatic function as evidenced by all of the following: total serum bilirubin ≤1.5× the upper limit of normal (ULN) and a prothrombin time within 2 seconds above the ULN and aspartate aminotransferase (AST)/alanine aminotransferase (ALT) must be ≤2.5× ULN.
- Subjects must have all of the following: platelet count >100000/μL, hemoglobin ≥10.0 g/dL, white blood cell (WBC) count >2000/μL, absolute neutrophil count (ANC) ≥1.5×10^9^/L, and serum creatinine ≤1.5 mg/dL unless the measured creatinine clearance was >40 mL/min/1.73 m^2^.
- Women of childbearing potential with a negative serum pregnancy test (β-human chorionic gonadotropin) within 7 days prior to eligibility.
- Provided signed informed consent.

*Exclusion criteria*

Subjects who met any of following criteria were to be excluded from study entry:

- Subjects with Child-Pugh Class B or C cirrhosis or with evidence of portal hypertension by history, endoscopy, or radiologic studies.
- Subjects who were unable to undergo general anesthesia for any reason. Those with New York Heart Association active cardiac conditions. An evaluation of risk was to occur for those with functional classification II, III, or IV. This included, but was not limited to, the following conditions: unstable coronary syndrome (unstable or severe angina or myocardial infarction within 6 months prior to Screening), worsening or new-onset congestive heart failure, significant arrhythmias, or severe valvular disease.
- History or evidence of clinically significant pulmonary disease that precluded the use of general anesthesia.
- Women of childbearing potential, i.e., fertile meaning not permanently sterilized and having had a menstrual period within the past 12 months, unable to undergo hormonal suppression to avoid menstruation during treatment.
- Women of childbearing potential and fertile males (not permanently sterile by bilateral orchidectomy) unwilling or unable to use highly effective contraception method for consent to at least 6 months after the last administration of study treatment (e.g., combined hormonal contraception, progestogen-only hormonal contraception, intrauterine device, intrauterine hormone-releasing system, bilateral tubal occlusion, vasectomized partner, or sexual abstinence).
- Females who were pregnant or breastfeeding.
- Subjects taking immunosuppressive drugs; however, oral corticosteroids ≤10 mg/day were allowed.
- Subjects unable to be temporarily removed from chronic anticoagulation therapy.
- Subjects with active bacterial infections with systemic manifestations (malaise, fever, or leukocytosis) were not eligible until completion of appropriate therapy.
- Subjects with severe allergic reaction to iodine contrast, which cannot be controlled by premedication with antihistamines and steroids.
- Subjects with a history of or known hypersensitivity to melphalan or the components of melphalan/HDS.
- Subjects with a latex allergy.
- Subjects with a history of hypersensitivity to heparin or the presence of heparin-induced thrombocytopenia.
- Subjects with a history of bleeding disorders or evidence of intracranial abnormalities that would put them at risk for bleeding with anticoagulation (e.g., strokes or active metastases).
- Subjects with a history of gastrinoma. Note: For subjects with a history of liver surgery or major vasculature surgery, a CT scan or MR angiogram was required during Screening to assure that the subject did not have hepatic vasculature incompatible with perfusion, hepatofungal flow in the portal vein, or known unresolved venous shunting.
- Known varices at risk of bleeding, including medium or large esophageal or gastric varices or active peptic ulcer.
- Subjects with prior Whipple’s procedure.
- Subjects with brain metastases or presence of other intracranial lesions at risk for bleeding by history or baseline radiologic imaging.
- Subjects with an active liver infection, including hepatitis B and hepatitis C infection.
- Subjects with anti-hepatitis B core antibody positive or hepatitis B surface antigen but DNA negative were exception(s).
- Uncontrolled endocrine disorders including diabetes mellitus, hypothyroidism, or hyperthyroidism.
- Received any investigational agent for any indication within 30 days prior to the first treatment.
- Not recovered from side effects of prior therapy to ≤Grade 1 (according to National Cancer Institute [NCI] Common Terminology Criteria for Adverse Events [CTCAE] v.4.03). Certain side effects that were unlikely to develop into serious or life-threatening events (e.g., alopecia) are allowed at >Grade 1.
- Cancers other than UM for which the subject was currently under treatment or still deemed to be not cancer-free.

***Study treatment: melphalan/Hepatic Delivery System (melphalan/HDS)***

The melphalan/HDS is a co-packaged drug-device combination product. Melphalan is a bifunctional alkylating agent with broad efficacy as an anticancer chemotherapeutic agent against various tumor histologies. It is well-studied and has limited liver toxicity, a high hepatic extraction rate, a short half-life, and an immediate apoptosis effect on tumor cells.^1,2^ The HDS consists of an extracorporeal hemofiltration circuit (EFC), an infusion catheter to deliver melphalan to the hepatic artery, and a femoral access set. The EFC is a closed circuit of catheters that lowers the melphalan concentration in the hepatic venous blood before it is returned to the systemic circulation. The EFC includes a double-balloon catheter placed in the retro-hepatic inferior vena cava to isolate the hepatic venous blood from the systemic circulation, a hemofiltration circuit including hemofiltration cartridges to adsorb melphalan and a venous return line. This procedure of separating the liver with the double-balloon catheter, perfusion with melphalan, and hepatic venous blood filtration is called percutaneous hepatic perfusion.^3^

***Dose and schedule of melphalan/HDS treatment***

Patients with unresectable hepatic-dominant metastatic uveal melanoma (UM) received melphalan (3.0 mg/kg ideal body weight; maximum dose: 220 mg for a single treatment) once every 6-8 weeks for a maximum of 6 cycles, with an acceptable delay of 2 weeks (i.e., 8 weeks in total) between cycles to allow for recovery from melphalan-related toxicity. Melphalan dose was calculated as shown in the table below.

***Ideal body weight calculation***

| **Gender** | **Height** | **Ideal body weight** |
| --- | --- | --- |
| Male | ≥152 cm | 52 kg + 0.75 kg/cm of height >152 cm |
|  | <152 cm | 52 kg – 0.75 kg/cm of height <152 cm |
| Female | ≥152 cm | 49 kg + 0.67 kg/cm of height >152 cm |
|  | <152 cm | 49 kg – 0.67 kg/cm of height <152 cm |

Each treatment cycle consisted of 6 weeks with a window of ±2 weeks. The second to sixth treatments were not administered if one of the following criteria applied:

- Hepatic disease progression
- Extrahepatic disease progression
- Treatment delay of 2 weeks (i.e., 8 weeks after the last melphalan/HDS treatment) due to prolonged toxicity

Tumor response was assessed every 12 weeks (±2 weeks) until disease progression. Patients with hepatic or extrahepatic progressive disease were discontinued from study treatment, and all patients were followed until death. The maximum duration of the study treatment phase for any patient was 12 months. Once a patient finished their treatments, they entered the follow-up period. If the patient had not progressed after their last treatment cycle, disease assessments were continued every 12 weeks (±2 weeks) until disease progression was documented. Meanwhile, if a patient had progressed after their last treatment cycle, their follow-up for survival status was done either in person or by phone every 3 months until death.

The dose of melphalan was to be reduced to 2.0 mg/kg for subsequent cycles if any of the following toxicities was observed:

- Grade 4 neutropenia lasting >5 days despite granulocyte growth factor support or Grade 3 or higher febrile neutropenia with granulocyte growth factor support.
- Grade 4 thrombocytopenia lasting >5 days or Grade 3 thrombocytopenia associated with medically significant bleeding.

**Meta-analysis of historical data and sample size considerations**

The meta-analysis of historical data included as a part of the information package for the US FDA, included data from 476 patients in 16 publications on both single agent and combination therapy response rates for immunotherapy treatments (e.g., checkpoint inhibitors) in patients with UM.

*Individual Study Rates*

Based on the data abstracted from each publication, the objective response rate (ORR) was calculated for each publication along with the 95% confidence interval (CI) of the estimates. When a publication reported a 0% event rate, an adjusted rate was calculated for the event. The adjusted rate for studies with no events was calculated as follows:

Rate=1/(2*n +2)

Where n=sample size in the article.

Statistical analyses utilized the logit transformation (log odds) of the event rates. The 95% CI was converted from the upper and lower limits of the Logit event rate and from them derived the upper and lower limits of the event rate as follows:

LogitEventRateLower=LogitEventRate - 1.96* LogitEventRateSE

LogitEventRateUpper=LogitEventRate + 1.96* LogitEventRateSE

EventRateLower=Exp(LogitEventRateLower) / (Exp(LogitEventRateLower) + 1)

EventRateUpper=Exp(LogitEventRateUpper) / (Exp(LogitEventRateUpper) + 1)

*Pooled Rates Across Studies*

Pooled rates across studies for ORR were calculated based on both a random effects model and a fixed effects model. The random effects model utilized the approach of DerSimonian and Laird (1986).^4^ The results of the random effect model are presented and discussed in this report and the results of the fixed effect model are available upon request.

*Testing Heterogeneity of the Studies*

Heterogeneity in effect size was formally examined using 2 statistics. The first statistic is the Q statistic.^4^ Q is the weighted sum of squares (WSS) on a standardized scale. As a standard score, it can be compared with the expected WSS on the assumption that all studies share a common effect. Hence, the Q statistic yields a test of whether all studies share a common effect size; the p-value of this test is reported. The null hypothesis for this test is that the effect sizes across all studies are homogenous (similar). Thus, a small p-value would indicate significant heterogeneity of effect sizes.

The second statistic is the I^2^ statistic.^5^ I^2^ is the proportion of observed dispersion (of effect sizes across studies) that is real, rather than spurious. It is not dependent on the scale and it is expressed as a ratio with a range of 0% to 100%. For I^2^ value below 60%, heterogeneity was considered moderate; for I^2^ value above 60%, heterogeneity was considered large. If I^2^ is near zero, then almost all the observed variance is spurious.

*Analysis Software*

Comprehensive Meta-Analysis (CMA) software version 2.2 was used to perform the analysis. The analysis results were copied and pasted to an excel file prior to formatting for this report. CMA version 2.2 was developed by Borenstein et al. at Biostat, 14 North Dean Street, Englewood, NJ 07631 (www.Meta-Analysis.com). Computation details can be found in Introduction to Meta-Analysis by M. Borenstein, L.V. Hedges, J.P.T. Higgins, H.R. Rothstein© 2009, John Wiley & Sons, Ltd.^5^

*Results*

Based on the data identified in 16 publications on response rates for immunotherapy treatments,^6-21^ both single agent and combination therapy, in patients with UM, the ORR was calculated for study along with the 95% CI and is presented in the table below. Across all 16 references, a total of 17 events (complete response + partial response) were reported in 476 patients. The pooled ORR estimate (a weighted mean of the observed ORR) was 0.055 and 95% CI was 0.036 to 0.083.

**Meta-analysis - Reported and estimated objective response rates and 95% confidence interval**

| Study no. | Reference ID^a^ | No. of events^b^ | Sample size | Event rate | 95% confidence interval | |
| --- | --- | --- | --- | --- | --- | --- |
|  |  |  |  |  | Lower limit | Upper limit |
| 1 | Luke et al. 2013^6^ | 2 | 39 | 0.051 | 0.013 | 0.183 |
| 2 | Maio et al. 2013^7^ | 4 | 82 | 0.049 | 0.018 | 0.123 |
| 3 | Zimmer et al. 2015^8^ | 0 | 53 | 0.009 | 0.001 | 0.131 |
| 4 | Danielli et al. 2012^9^ | 0 | 13 | 0.036 | 0.002 | 0.384 |
| 5 | Wiater et al. 2013^10^ | 0 | 9 | 0.050 | 0.003 | 0.475 |
| 6 | Khattak 2013^11^ | 0 | 5 | 0.083 | 0.005 | 0.622 |
| 7 | Rozeman 2017^12^ | 0 | 19 | 0.025 | 0.002 | 0.298 |
| 8 | Karydis et al. 2016^13^ | 2 | 25 | 0.080 | 0.125 | 0.269 |
| 9 | Kottschade et al. 2016^14^ | 3 | 8 | 0.375 | 0.009 | 0.715 |
| 10 | Algazi et al. 2016^15^ | 2 | 56 | 0.036 | 0.018 | 0.132 |
| 11 | Heppt 2017^16^ | 4 | 86 | 0.047 | 0.002 | 0.117 |
| 12 | Van der Kooij et al. 2017^17^ | 0 | 17 | 0.028 | 0.003 | 0.322 |
| 13 | Bender 2017^18^ | 0 | 15 | 0.031 | 0.001 | 0.350 |
| 14 | Gonzalez-Cao et al. 2017^19^ | 0 | 8 | 0.056 | 0.001 | 0.505 |
| 15 | Piperno-Neumann et al. 2016^20^ | 0 | 21 | 0.023 | 0.0 | 0.277 |
| 16 | KEYTRUDA SmPC 2018^21^ | 0 | 20 | 0.024 | 0.0 | 0.287 |
| Pooled (n = 16) | | 17 | 476 | 0.055 | 0.036 | 0.083 |
| Study heterogeneity statistics^c^ | | Q = 14.46, df(Q) = 15, *P* = .491, I^2^ < .001 | | | | |
| Pooled without Study 9 (n = 15) | | 14 | 468 | 0.044 | 0.028 | 0.068 |
| Study heterogeneity statistics^c^ | | Q = 3.24, df(Q) = 14, *P* = .999, I^2^ < .001 | | | | |
| ^a^ 16 publications on response rates for immunotherapy treatments (single agent and combination therapy) in patients with ocular melanoma.  ^b^ Event rate was estimated using 1/(2*n÷2) when the reported event was zero.  ^c^ Null hypothesis for Q test: effect sizes across all studies are homogenous. Thus, a small *P* value would indicate significant heterogeneity of effect sizes. If I^2^ is near zero, then almost all the observed variance is spurious. | | | | | | |

## **References**

1. Shah GL, Boelens JJ, Carlow D, et al: Population pharmacokinetics of melphalan in a large cohort of autologous and allogeneic hematopoietic cell transplantation recipients: Towards individualized dosing regimens. Clin Pharmacokinet 2022;61(4):553-563.
2. ALKERAN^®^ (melphalan hydrochloride) for Injection [package insert]. GlaxoSmithKline 2011. U.S. Food and Drug Administration website. Available at [label (fda.gov)](https://www.accessdata.fda.gov/drugsatfda_docs/label/2011/020207s016lbl.pdf).
3. Modi S, Gibson T, Vigneswaran G, et al: Chemosaturation with percutaneous hepatic perfusion of melphalan for metastatic uveal melanoma. Melanoma Res 2022;32(2):103-111.
4. DerSimonian R, Laird N: Meta-analysis in clinical trials. Control Clin Trials 1986;7(3):177-188.
5. Borenstein M, Hedges LV, Higgins JPT, et al: Introduction to Meta-analysis (Chapter 16). Chichester, West Sussex, U.K.; Hoboken: John Wiley & Sons; 2009.
6. Luke JJ, Callahan MK, Postowet MA, et al: Clinical activity of ipilimumab for metastatic uveal melanoma: A retrospective review of the Dana-Farber Cancer Institute, Massachusetts General Hospital, Memorial Sloan-Kettering Cancer Center and University Hospital of Lausanne experience. Cancer 2013;119(20):3687-3695.
7. Maio M, Danielli R, Chiarion-Sileni V, et al: Efficacy and safety of ipilimumab in patients with pre-treated, uveal melanoma. Ann Oncol 2013;24(11):2911-2915.
8. Zimmer L, Vaubel J, Mohr P, et al: Phase II DeCOG-study of ipilimumab in pretreated and treatment-naïve patients with metastatic uveal melanoma. PLoS One. 2015;10(3):e0118564.
9. Danielli R, Ridolfi R, Chiarion-Sileni V, et al: Ipilimumab in pretreated patients with metastatic uveal melanoma: Safety and clinical efficacy. Cancer Immunol Immunother 2012;61(1):41-48.
10. Wiater K, Switaj T, Mackiewicz J, et al: Efficacy and safety of ipilimumab therapy in patients with metastatic melanoma: A retrospective multicenter analysis. Contemp Oncol (Pozn) 2013;17(3):257-262.
11. Khattak MA, Fisher R, Hughes P, et al: Ipilimumab activity in advanced uveal melanoma. Melanoma Res 2013;23(1):79-81.
12. Rozeman EA, Fanchi L, van Akkooi ACJ, et al: (Neo) adjuvant ipilimumab + nivolumab (IPI+NIVO) in palpable stage 3 melanoma – updated relapse free survival (RFS) data from the OpACIN trial and first biomarker analysis. Ann Oncol 2017;28 (suppl_5):v428-v448.
13. Karydis I, Chan PY, Wheater M, et al: Clinical activity and safety of pembrolizumab in ipilimumab pre-treated patients with uveal melanoma. Oncoimmunology 2016;5(5):e1143997.
14. Kottschade LA, McWilliams RR, Markovic SN, et al: The use of pembrolizumab for the treatment of metastatic uveal melanoma. Melanoma Res 2016;26(3):300-303.
15. Algazi AP, Tsai KK, Shoushtari AN, et al: Clinical outcomes in metastatic uveal melanoma treated with PD-1 and PD-L1 antibodies. Cancer 2016;122(21):3344-3353.
16. Heppt MV, Steeb T, Schlager JG, et al: Immune checkpoint blockade for unresectable or metastatic uveal melanoma: A systematic review. Cancer Treat Rev 2017;60:44-52.
17. van der Kooij MK, Joosse A, Speetjens FM, et al: Anti PD1 treatment in metastatic uveal melanoma in the Netherlands. Acta Oncol 2017;56(1):101-103.
18. Bender C, Enk A, Gutzmer R, and Hassel JC: Anti-PD-1 antibodies in metastatic uveal melanoma: A treatment option? Cancer Med 2017;6(7):1581-1586.
19. González-Cao M, Arance A, Piulats JM, et al: Spanish Melanoma Group. Pembrolizumab for advanced melanoma: Experience from the Spanish Expanded Access Program. Clin Transl Oncol 2017;19(6):761-768.
20. Piperno-Neumann S. Diallo A, Etienne-Grimaldi MC, et al: Phase II trial of bevacizumab in combination with temozolomide as first-line treatment in patients with metastatic uveal melanoma. Oncologist 2016;21(3):281-282.
21. KEYTRUDA (pembrolizumab) Summary of Product Characteristics. Merck Sharp & Dohme B.V. Available at [Keytruda, INN-pembrolizumab (europa.eu)](https://www.ema.europa.eu/en/documents/product-information/keytruda-epar-product-information_en.pdf).
